# Ab-initio study of ReCN in the bulk and as a new two dimensional material

**DOI:** 10.1038/s41598-017-03072-6

**Published:** 2017-06-05

**Authors:** J. Guerrero-Sánchez, Noboru Takeuchi, A. Reyes-Serrato

**Affiliations:** Centro de Nanociencias y Nanotecnología, Universidad Nacional Autónoma de México, Apartado Postal 14, Ensenada Baja California, Código Postal, 22800 Mexico

## Abstract

First principles total energy calculations have been applied to describe the ReCN bulk structure and the formation of ReCN monolayers and bilayers. Results demonstrate a strong structural rearrangement in the monolayer due to a reduced dimension effect: an increase in the lattice parameter, accompanied with the contraction of the distance between the C and N planes. On the other hand, a ReCN bilayer has structural parameters similar to those of the bulk. Surface formation energies show that the monolayer is more stable than bilayer geometries. Although bulk ReCN shows a semiconductor behavior, the monolayer ReCN presents a metallic behavior. This metallic character of the ReCN monolayer is mainly due to the d-orbitals of Re atoms.

## Introduction

Graphene is a well-known 2D material with outstanding properties, making it one of the most studied materials. It can be obtained by exfoliation^1^, or by epitaxial growth on different substrates^2, 3^. It presents physical properties such as substrate-induced band gap opening^3^, a half-metallic behavior^4^, a tunable gap^5^, remarkable electronic mobility^6^, and a topological insulator behavior^7^. These properties make graphene suitable for many technological applications^8^. For example, it may be used as photodetector^9^, optical modulator^10^, as key constituent in solar cells, in light emitting devices, and in ultrafast lasers^11^, as well as in flexible optoelectronic devices^12^, among other specific applications^13, 14^.

Since graphene gained such attention, many research groups started to look for new 2D materials with equally interesting properties. As consequence, several 2D materials have been found: silicene^15^, germanene^16^, and tri-layer transition metal dichalcogenides^17–19^ are some examples. These 2D materials have excellent properties, which may be exploited to construct new generation devices, or to form heterostructures with graphene to expand its well established applications as well as to tune its unprecedented properties^20–22^. Recently, a new two dimensional semiconductor has been obtained: black phosphorene. This novel material has been proposed as a strong competitor to graphene since it has a semiconductor behavior, with a band gap that can be modulated by increasing the number of layers^23^. It also can be highly strained without losing its semiconductor character, making it suitable for applications in flexible electronic devices^24^. It can change from direct to indirect gap by tensile strain^25^. Furthermore, its high carrier mobility makes it a potential material for the channel in the construction of electronic and optoelectronic devices^26–28^.

Keeping in mind the interest that 2D materials have generated in the past years, we have turned our attention to the ReCN compound. This is a super hard material with an orthogonal bulk structure with two ReCN tri-layers in the unit cell^29^. Since two consecutive tri-layers are not bonded by weak van der Waals forces, it is not possible to obtain 2D ReCN by exfoliation. However, it surely may be grown by techniques such as molecular beam epitaxy, chemical vapor deposition, spray pyrolysis or some other chemical or physical growth techniques. To explore this material deeper, we have carried out first principles calculations to characterize the structural and electronic properties of ReCN in bulk and as a 2D material.

## Method

Calculations have been carried out using the density functional theory as implemented in the PWscf code of the Quantum ESPRESSO package^30^. The Kohn-Sham states have been expanded in plane waves with a kinetic energy cutoff of 30 Ry, while the charge density cutoff was set to 240 Ry. The generalized gradient approximation with the Perdew-Burke-Ernzerhof parametrization^31^ has been used to treat the non-classical exchange and correlation energy. Vanderbilt ultra-soft pseudopotentials were used to replace the effect of core electrons. To evaluate the Brillouin zone integrations we have used a **k**-points mesh of 4 × 4 × 1^32^.

To begin with the calculations, we have optimized the ReCN bulk structure in a tetragonal structure, with space group P63mc (186)^29^. The monolayer has been simulated by taking only a Re-C-N unit with an empty space large enough (~15 Å) to account for the surface effects in the 2D system as well as to eliminate undesirable interactions between adjacent monolayers. A similar procedure has been used to simulate the bilayers, with two different configurations.

## Results and Discussion

### ReCN bulk structure

**Figure 1:**
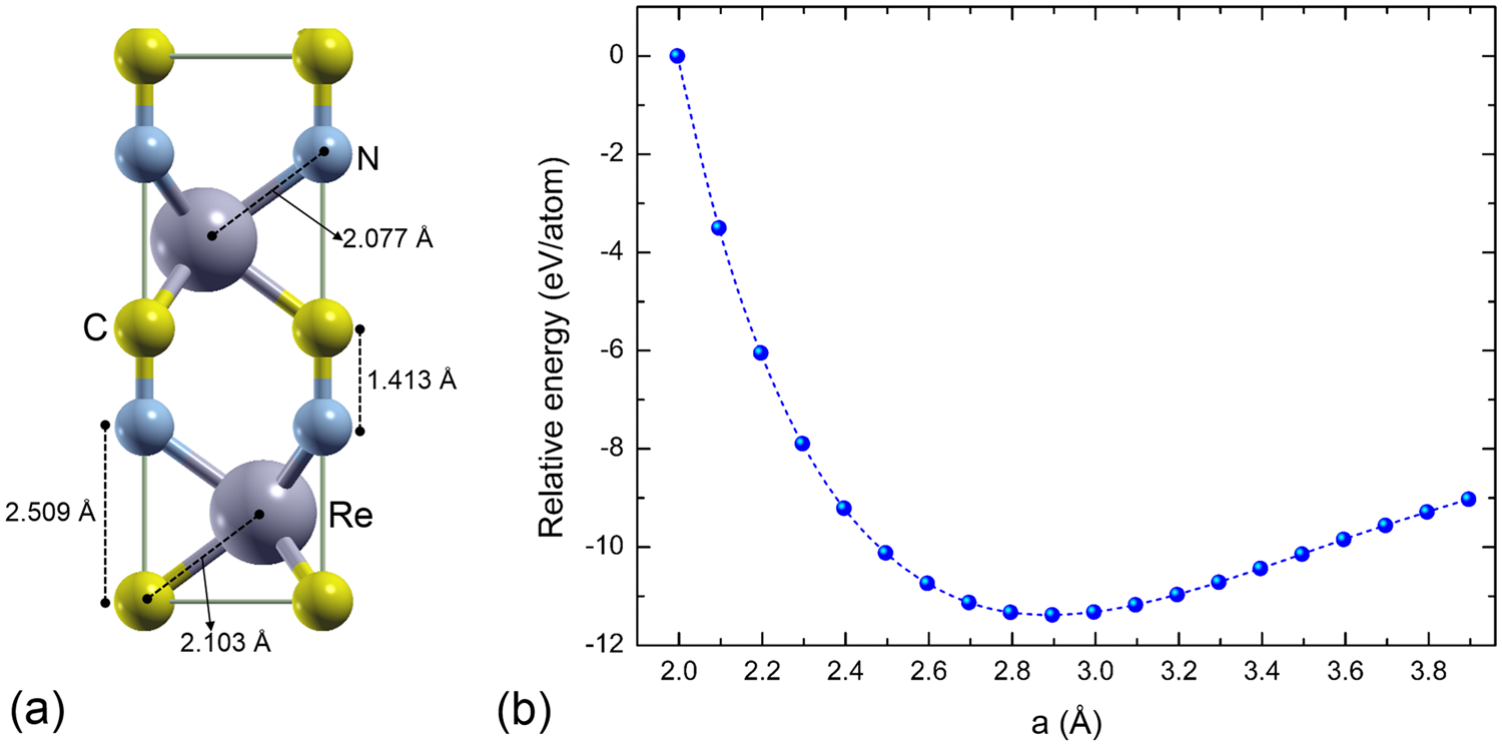
(**a**) ReCN bulk structure, and (**b**) Total energy as function of lattice parameter for bulk ReCN.

Previous calculations of bulk ReCN have found a stable tetragonal structure with lattice parameters: a = 2.897 Å and c = 7.851 Å^29^, and a semiconductor behavior with a calculated energy gap of ~0.6 eV^29^. Before studying the ReCN 2D system, we have reproduced the ReCN bulk structure, as shown in Fig. 1(a). Our fully optimized lattice parameters are: a = 2.896 Å and c = 7.844 Å (Fig. 1(b)), which indicates good agreement with previous calculations^29^. The Re-C, Re-N, and C-N distances were found also in agreement with previously reported data, as seen in Table 1.

**Table 1:** Comparison between our calculated distances and previously reported data.

Structure	d_Re-C_ (Å)	d_Re-N_ (Å)	d_C-N_ (Å)
This work	2.103	2.077	1.413
Theory^29^	2.109	2.082	1.401

Figure 1(a) shows that in the ReCN structure, besides the C-N bonds formed by atoms of different tri-layer units, it is possible that the carbon and nitrogen atoms separated by a Re layer could be bonding. However, the atomic distance between C and N planes is 2.509 Å, indicating that the carbon and nitrogen atoms of those layers are not interacting directly.

### ReCN monolayer

**Figure 2:**
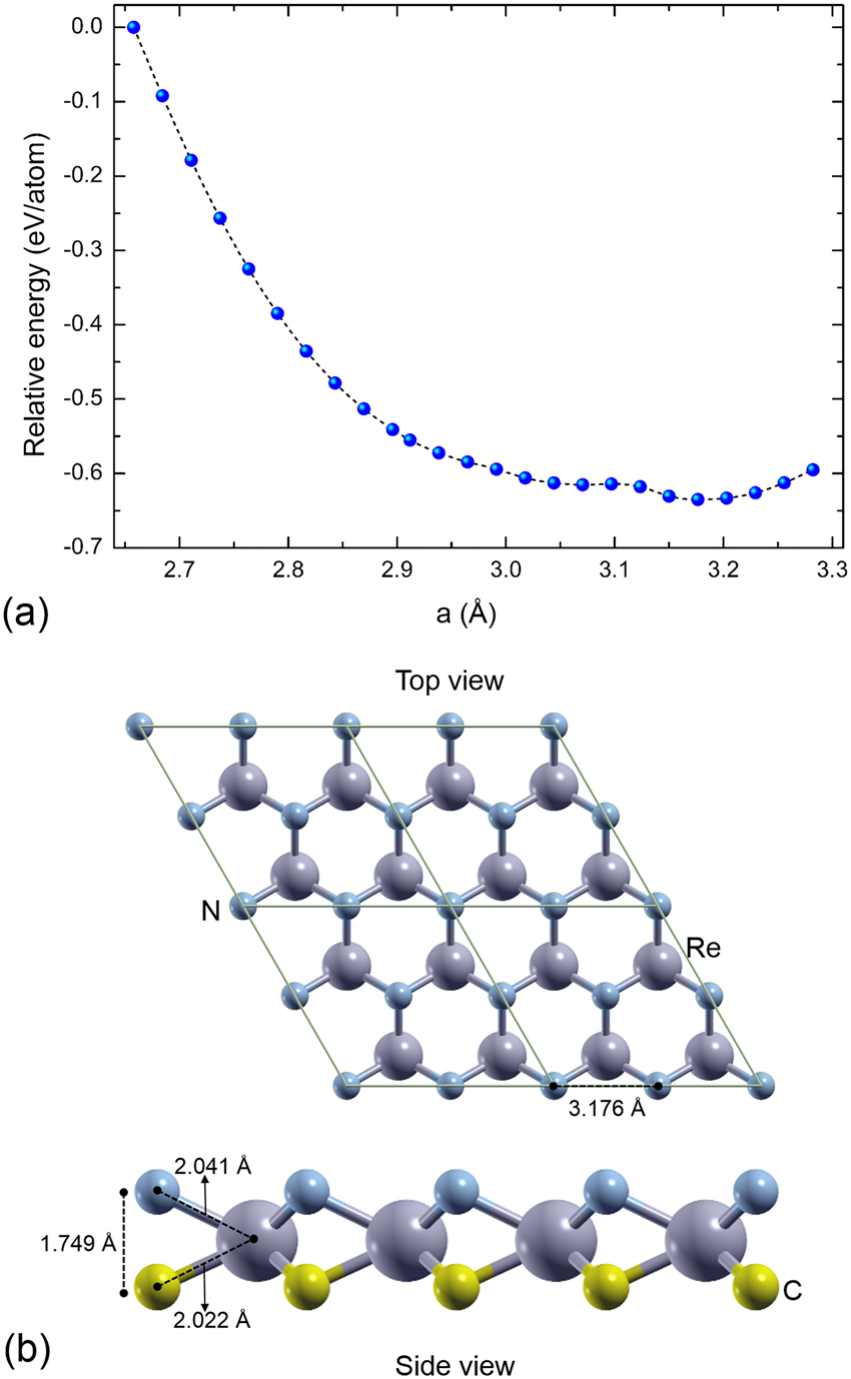
(**a**) Total energy as function of lattice parameter for a monolayer of ReCN. (**b**) Top and side view of the ReCN monolayer.

We have next studied the properties of a single ReCN monolayer. Since surface effects may generate structural changes, we have fully optimized the atomic structure of the monolayer. Figure 2(a) depicts the energy as function of the lattice parameter. Note that this tri-layer system presents a behavior similar to silicene, in which there is a high buckling metastable configuration and a more stable low buckling structure. In this case, the metastable structure has a lattice parameter (*a* = 3.071 Å) characterized by a C and N planes distance of 2.016 Å. In the low buckling structure, the lattice parameter has a value of 3.176 Å, which is around 9.67% larger than the lattice parameter in the bulk (Fig. 1(b)). With this modification in the lattice constant, the atomic distances change too. The Re-C and Re-N bonds decrease with respect to the values in the bulk by ~0.1 Å and ~0.05 Å, respectively (See Fig. 2(b)).

Different from bulk, in the ReCN monolayer there are no C-N bonds between atoms belonging to different tri-layer units, since in this case there in one monolayer only. However, the large structural changes in the monolayer reduce the distance between the C and N planes to 1.748 Å, a distance in which carbon and nitrogen atoms may be forming bonds.

### ReCN bilayers

**Figure 3:**
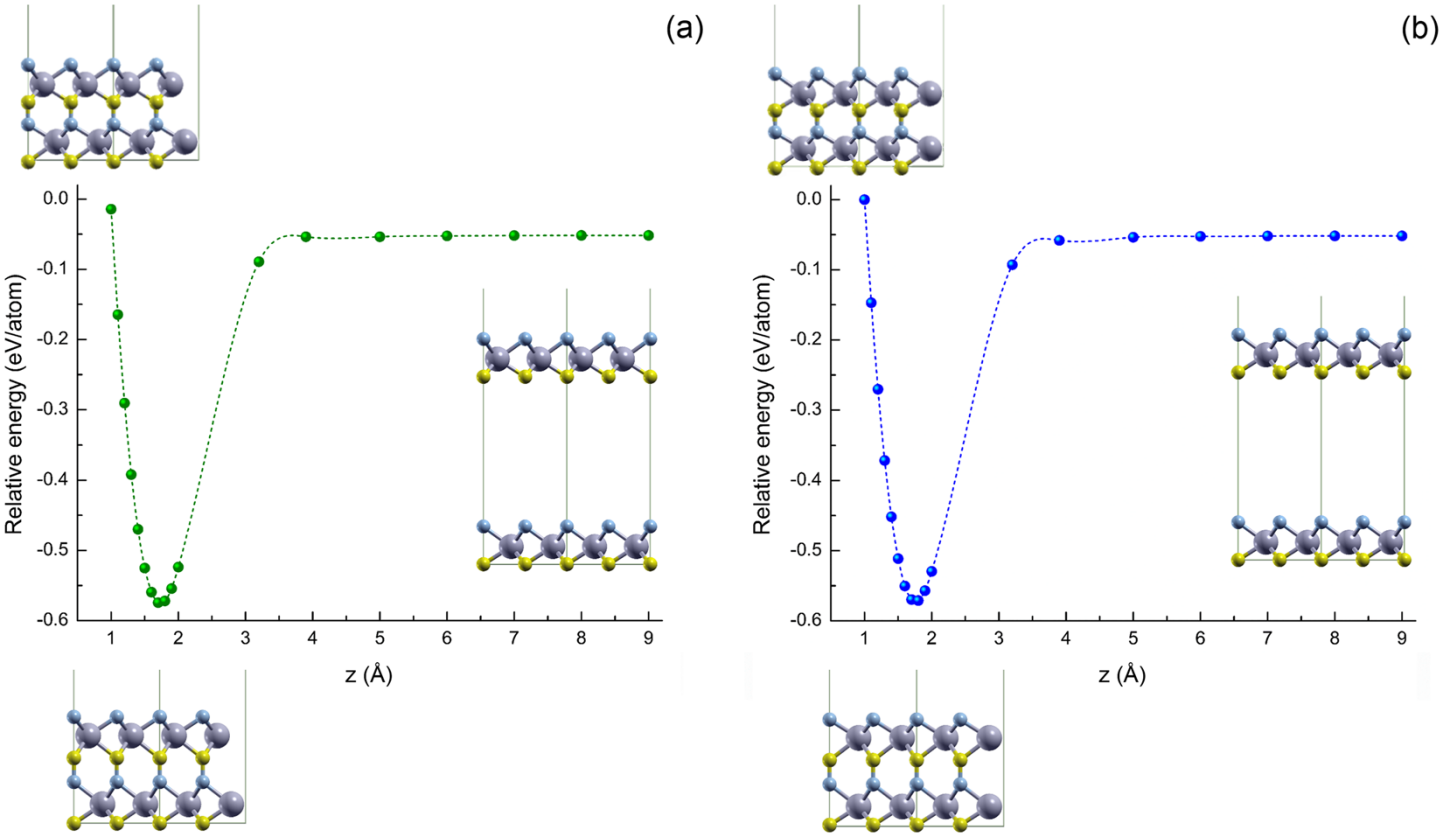
Interaction energy as function of the perpendicular distance between two ReCN monolayers. (**a**) Bulk-like stacking, and (**b**) on-top arrangement.

To analyze how two monolayers interact when they are near each other, we have plotted the interaction energy as function of the inter layer separation distance. Two different configurations were considered. In Fig. 3(a) the alignment of the monolayers is bulk like, while in Fig. 3(b) the two tri-layer units are on top of each other.

The behavior is similar in the two cases. At very small separation distances, there is a large repulsion, making the system unstable (as expected). As the monolayer-monolayer distance increases, the bilayer becomes stable in a structure with a C-N distance similar to the one in the bulk. Increasing the distance between monolayers, the separation becomes so large that there is no interaction between them and they behave as isolated monolayers.

**Figure 4:**
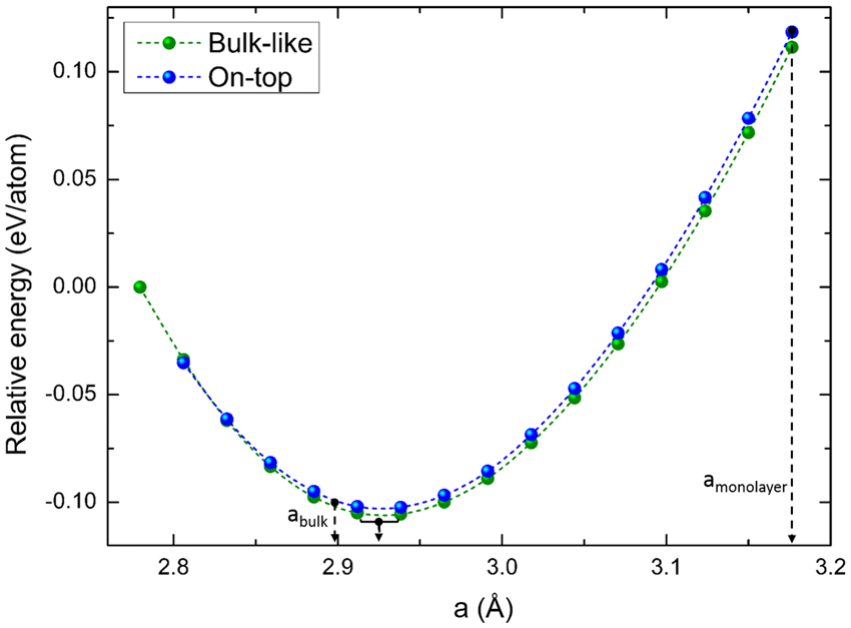
Total energy as function of lattice parameter for both bilayers.

At the most stable interaction distance, we have carried out a new structural optimization of the equilibrium lattice parameter. It is clear from Fig. 4 that the lattice parameter (a_bulk-like_ = a_on-top_ = 2.938 Å) decreases following a trend that should reach the bulk limit when the number of monolayers increase. It is also interesting to see that the bulk-like arrangement of the monolayers is slightly more stable than the on-top configuration.

A summary of the structural parameters of both geometries is presented in Table 2. From these results, we can see that both structures are structurally similar, and they differ only by the Re layer alignment. Also, the C and N planes distance (~2.380 Å) is the same in both configurations.

**Table 2:** Calculated average distances in the ReCN bilayers.

Structure	d_Re-C_ (Å)	d_Re-N_ (Å)	d_C-N_ (Å)
Bulk-like	2.077	2.069	1.405
On-top	2.076	2.071	1.414

### Stability analysis

We have considered several possible configurations with different number of atoms. To compare their relative stability, we use the surface formation energy formalism that depends on the chemical potential of each species of the system. It has the following form^33, 34^:

$${E}_{f}={E}_{model}-{E}_{reference}-\sum {n}_{i}{\mu }_{i}$$

where E_model_ is the energy of the system under study, E_reference_ is the reference energy, in this case, the one of a single ReCN monolayer, *n*_*i*_ is the number of extra atoms and ${\mu }_{i}$ the chemical potential of each species. Under this formalism, we can calculate the chemical potential as the total energy per atom of the most stable bulk structure. Then, ${\mu }_{{Re}}$ is obtained from a hexagonal lattice. As carbon has two allotropes, diamond and graphite, we have calculated the chemical potential of both structures. Finally, the N chemical potential is determined from an isolated N_2_ molecule. It is found that the monolayer configuration is more stable than any of the two bilayer configuration. This behavior is the same for both allotropes of carbon, as seen in Table 3.

**Table 3:** Calculated formation energies in eV/ReCN monolayer.

Model	Formation energy	
	C_graphite_	C_diamond_
Monolayer	0.00	0.00
Bilayer (Bulk-like)	0.705	0.307
Bilayer (On-top)	0.743	0.345

### Electronic properties

**Figure 5:**
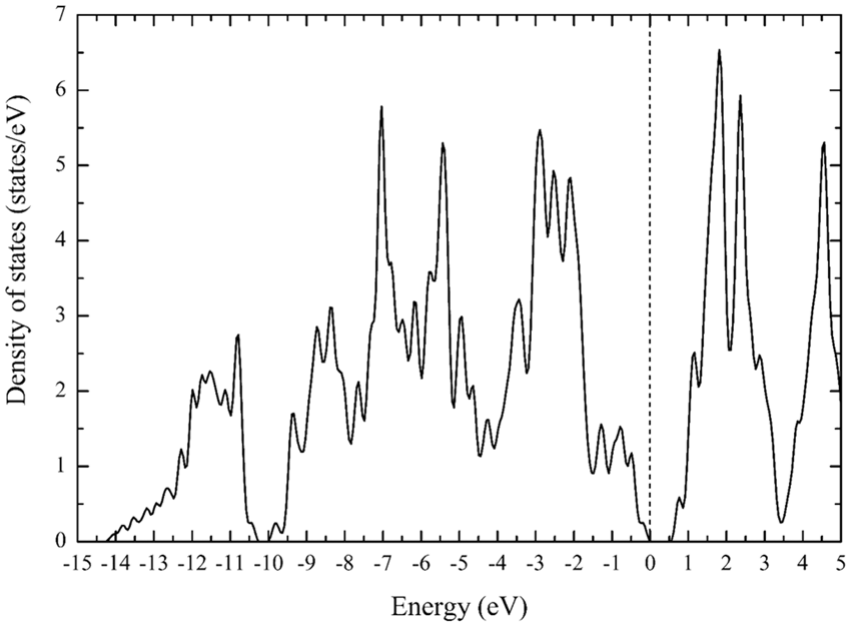
ReCN bulk density of states showing the semiconductor character. The reference energy was set at the energy of the last occupied state.

In this section, we report on the electronic properties of the different configurations that we have studied. Figure 5 shows the total density of states of bulk ReCN. A clear semiconductor behavior can be observed, with a calculated band gap of ~0.51 eV, in agreement with previous calculations^29^.

### Monolayer and bilayer density of states

**Figure 6:**
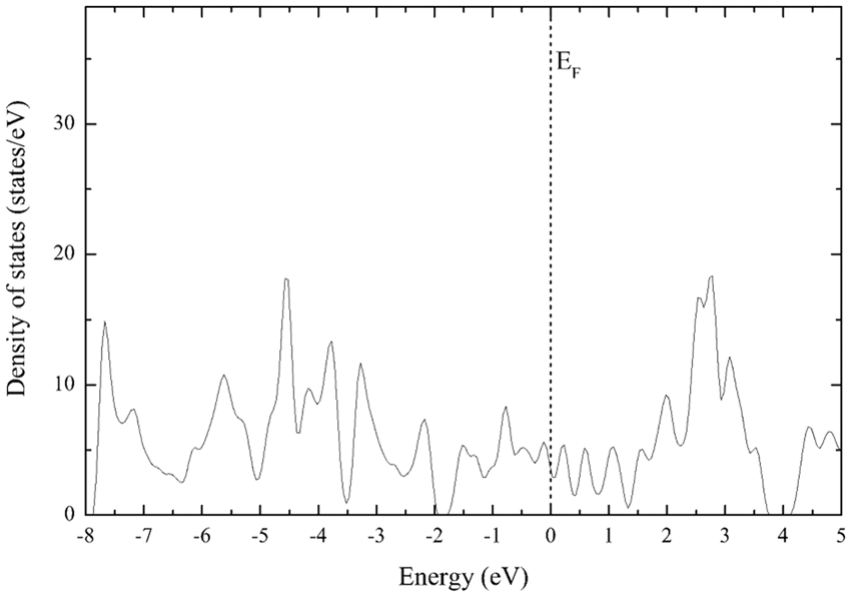
ReCN monolayer density of states. The reference energy was set at the Fermi level.

**Figure 7:**
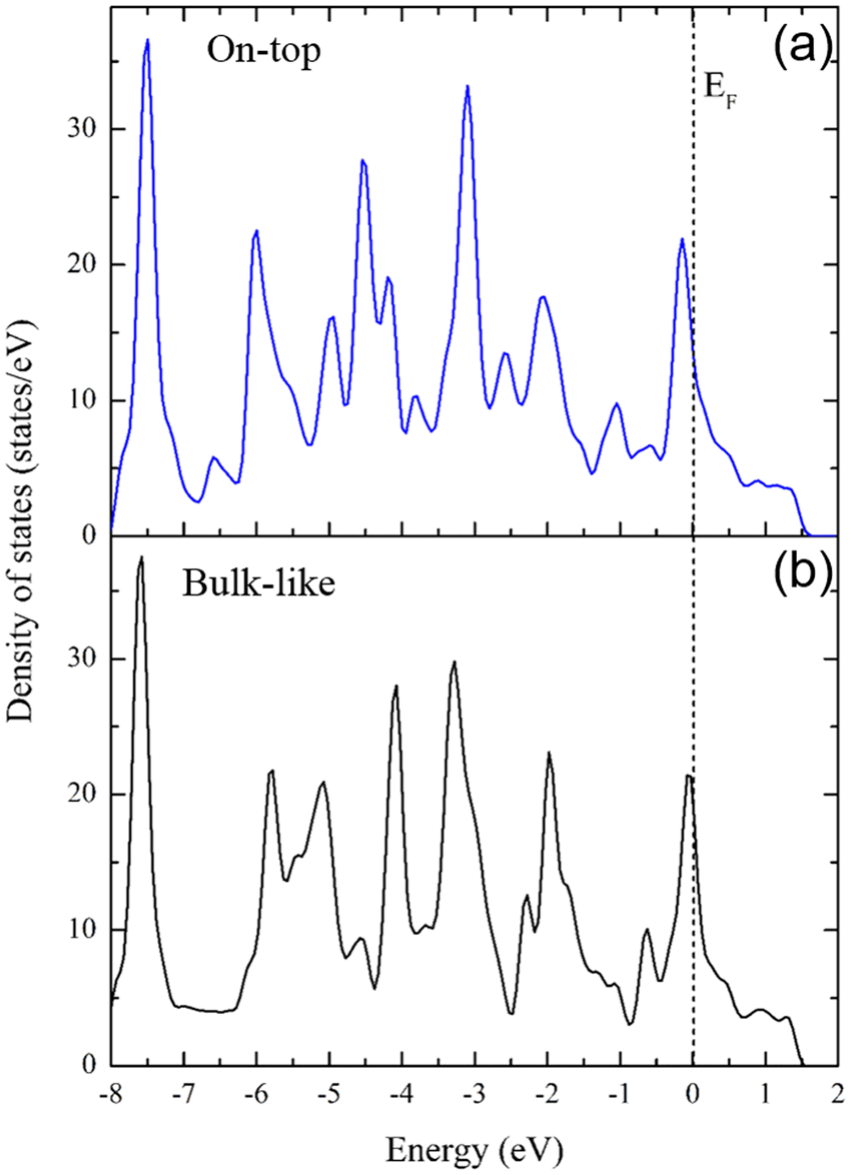
Density of states of (**a**) On-top and (**b)** Bulk-like arrangements. Reference energy was set to the Fermi level position.

Figure 6 shows the total density of states of an optimized single monolayer of ReCN. In this case, a high population of states is concentrated around the Fermi level, indicating a metallic behavior. Since bulk ReCN is semiconductor, we can say that the metallic behavior is due to the decrease in dimensionality. The total density of states of the two bilayers in an on top and bulk-like configurations are shown in Fig. 7(a) and (b) respectively. A clearly metallic behavior can be observed in both of them, with a peak at the Fermi level, indicating an electronic instability.

### Projected density of states of the stable ReCN monolayer

**Figure 8:**
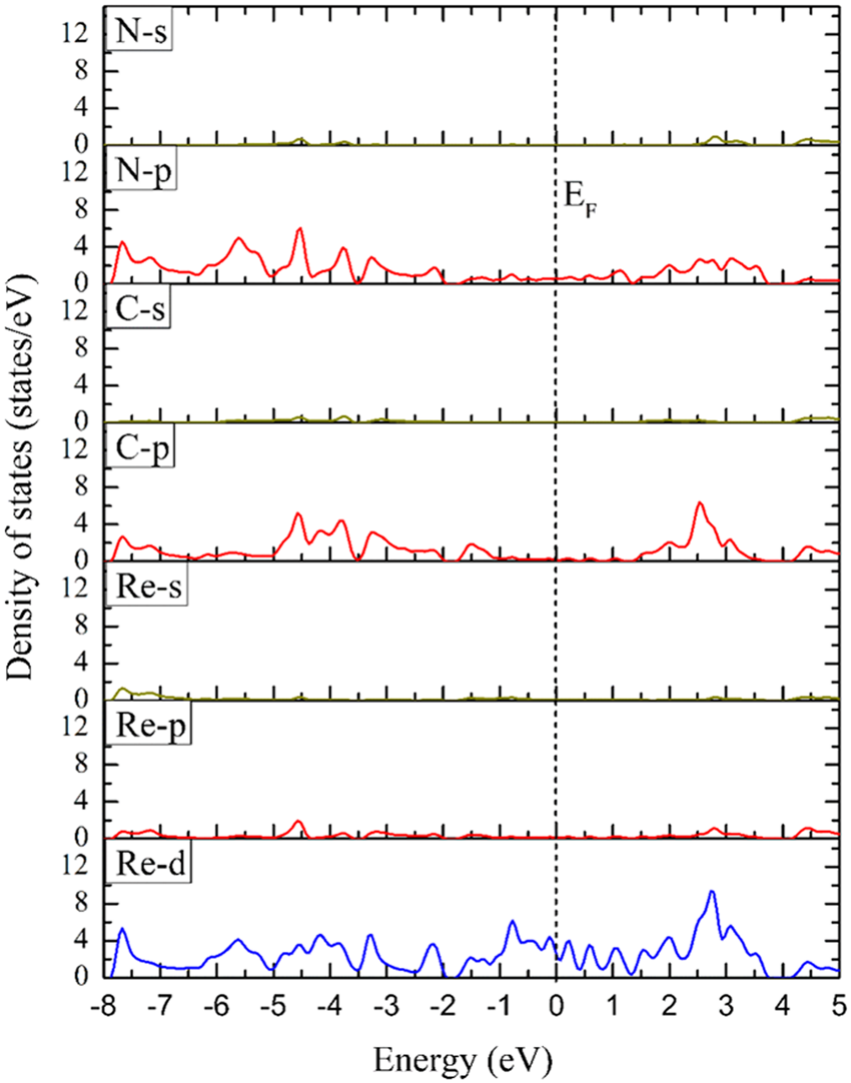
Projected density of states of the ReCN monolayer. Reference energy was set to the Fermi level.

We now concentrate in the stable ReCN monolayer system. In order to describe the orbitals which generate the metallic behavior in this structure, we plot the projected density of states (Fig. 8). It is clear that the Re-*d* orbitals have the most important contribution around the Fermi energy, being the main factor of the metallic behavior. The Re-*p*, Re-*s*, C-*s*, and N-*s* orbitals have a very small contribution to the density of states. On the other hand, the N-*p* and C-*p* orbitals have slightly contributions around the Fermi level. Their main contributions are for more negative and positive energies, as seen in Fig. 8.

**Figure 9:**
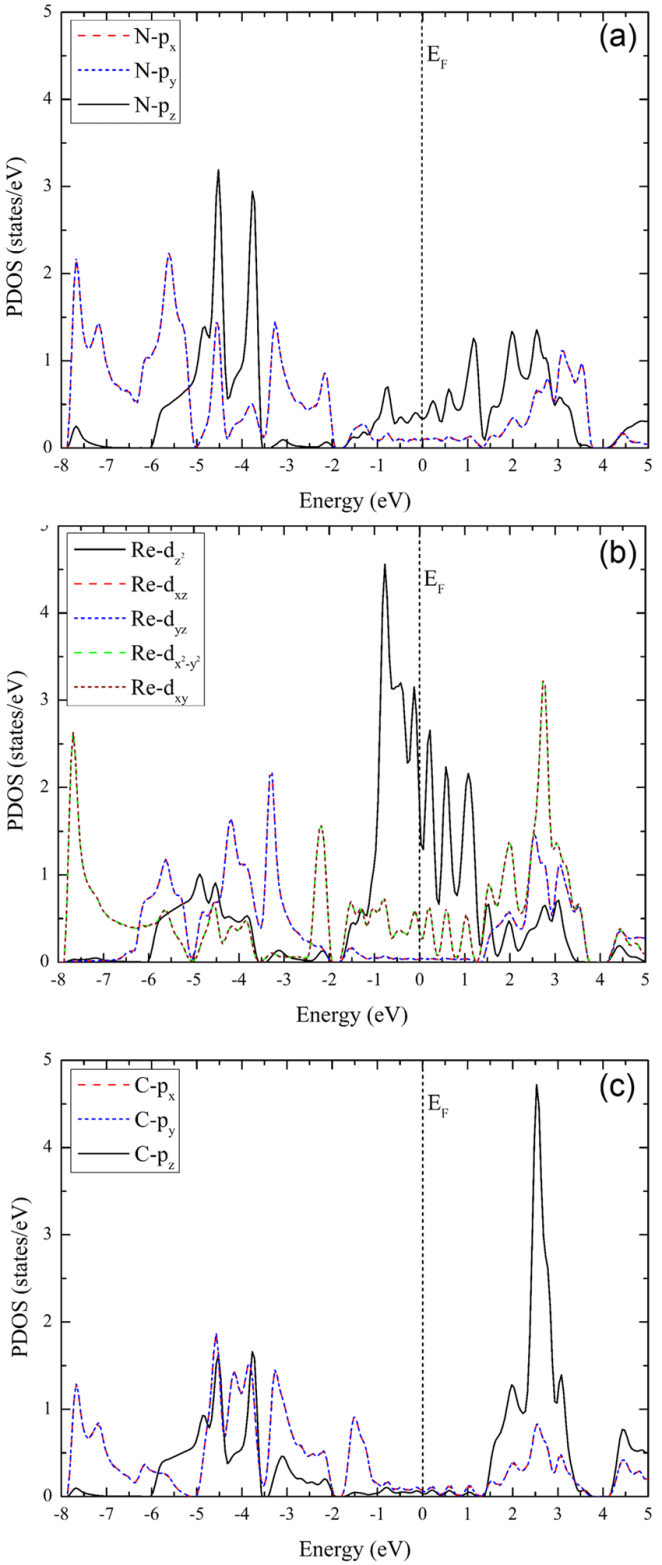
Orbital resolved partial density of states for: (**a**) N-p orbitals, (**b**) Re-d orbitals, and (**c**) C-p orbitals. The reference energy is the Fermi level.

Since the Re-*d*, C-*p* and N-*p* orbitals have the largest contributions to the density of states, we resolve them in to their components. In Fig. 9(a), we depict the resolved *p* orbitals of the N atom. In this case the N-*p*_*z*_ orbital has the main contribution around the Fermi level, with important contributions for more negative and positive energies too. The N-*p*_*x*_ and N-*p*_*y*_ orbitals are degenerated in all the range of energy. Figure 9(b) depicts the resolved Re-*d* orbitals. Here, the Re-*d*_*z*_^2^ component is the one with larger contribution around the Fermi energy, being the main factor of the metallic behavior. The remaining orbitals have large contributions for more negative and positive energies. Here, the Re-*d*_*xy*_, Re-*d*_*yz*_, Re-*d*_*x*_^2^_*−y*_^*2*^, and Re-*d*_*xy*_ orbitals are degenerated. Finally, the C-*p*_*z*_ orbital and the degenerated C-*p*_*x*_, and C-*p*_*y*_ orbitals have contributions for more negative and positive energies (Fig. 9(c)), with little contribution around the Fermi level. The degeneration of the orbitals in all atoms is a strong indicative of a highly symmetric and stable 2D structure.

### Charge density of the stable ReCN monolayer

**Figure 10:**
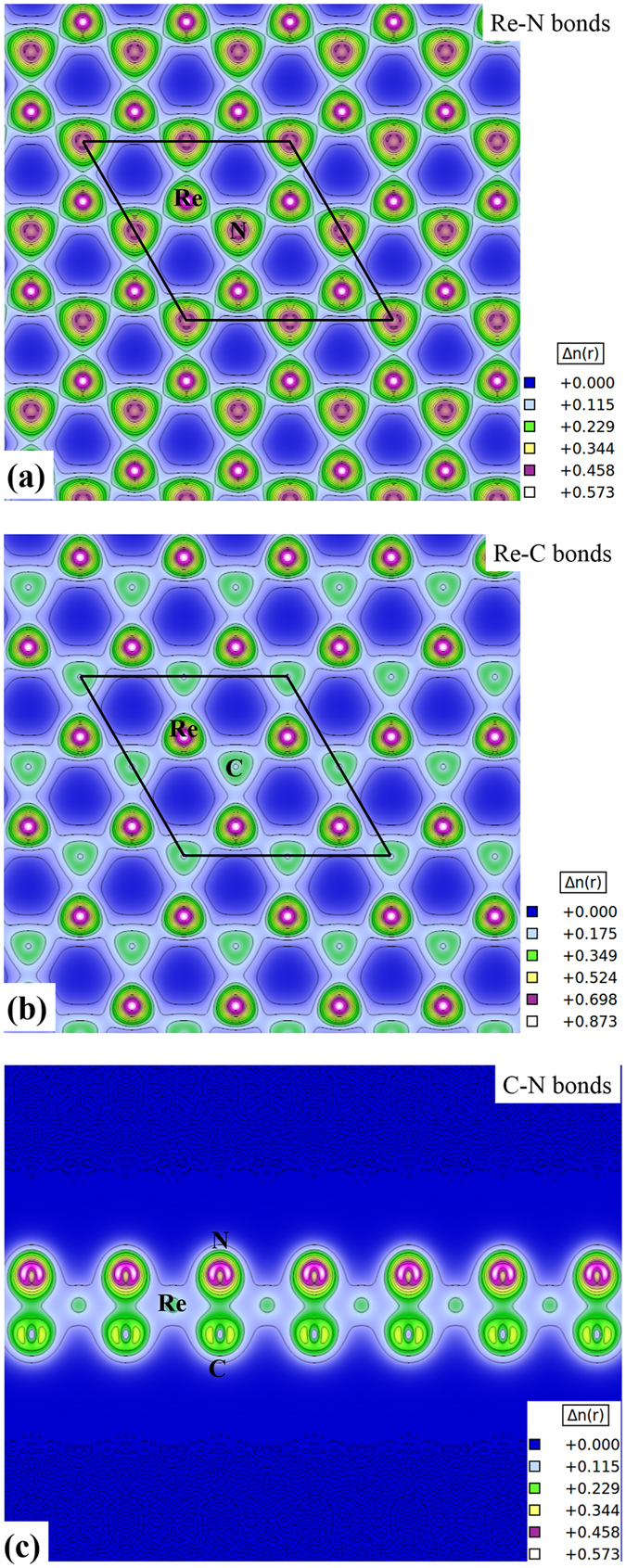
Charge density distributions for preferential planes in the ReCN monolayer: (**a**) Re-N bonds, (**b**) Re-C bonds, and (**c**) C-N bonds. Blue color corresponds to low charge density, and white color to high charge density concentration (value found only at the atomic sites).

To gain a better understanding of the Re-N, Re-C interactions and the possible bond formation between the C and N atoms, we plot the charge density in some preferential planes. Figure 10(a) depicts the charge density at the average height of the Re and N atoms. A large charge accumulation can be noted at the Re and N atoms position. It is also clear that Re and N atoms are sharing charge, forming covalent bonds. A similar behavior is observed between the Re and C atoms, but in this case the Re atoms have a larger charge accumulation than C atoms, indicating a polar covalent character (see Fig. 10(b)). Now, to analyze the possible formation of a C-N bond in the monolayer, we plot the charge density in the (010) plane. As mentioned in the previous section, the inter-atomic carbon nitrogen distances make it possible for the two atoms to form a bond. Figure 10(c) shows a strong interaction with a covalent character. Since N atoms are more electronegative than carbon atoms, there is more charge around the nitrogen atoms. This charge difference may be the driving force to the decrease in the distance between the C and N planes.

## Conclusions

Applying first principles calculations, we have described the structural and electronic properties of ReCN in the bulk, as a monolayer and as a bilayer. Results show an increase in the lattice parameter when a single ReCN monolayer is optimized. This effect is generated by the contraction of the distance between the C and N planes of the monolayer along the perpendicular direction. In the case of a bilayer two different layer stacking are possible. The lattice parameters of both of them are similar to the one of bulk. Surface formation energies show that the monolayer is more stable than the bilayer models. A semiconductor behavior has been found in bulk, whereas the monolayer and bilayer ReCN present metallic behavior, mainly induced by the *d*-orbitals of Re atoms. Charge density plots confirm the Re-N, Re-C and C-N covalent bonds driving to a strong electronic stability in the monolayer. This new single ReCN monolayer could be used to form heterostructures with other 2D materials such as graphene or phosphorene in new generation electronic devices. We hope our study motivate experimental studies of the ReCN monolayer.
